# Effect of peer-led outreach activities on injecting risk behavior among male drug users in Haryana, India

**DOI:** 10.1186/1477-7517-11-3

**Published:** 2014-02-04

**Authors:** Bindya Jain, Suneeta Krishnan, Sowmya Ramesh, Shrutika Sabarwal, Vijay Garg, Neeraj Dhingra

**Affiliations:** 1 Haryana State AIDS Control Society, C-15, Awas Bhawan, Sector-6, Panchkula, Haryana 134109 India; 2 St John Research Institute, Sarjapur Road, Bengaluru 560034 India; 3 Population Council, 1st Floor, 142 Golf Links, New Delhi 110003, India; 4 National AIDS Control Organization, Department of AIDS Control, Ministry of Health & Family Welfare, Government of India, New Delhi, India

**Keywords:** Injecting drug use, HIV risk behavior, Peer educator, Needle sharing

## Abstract

**Background:**

For the past two decades, there has been an enduring HIV epidemic among injecting drug users (IDUs) in India, and the Indian national AIDS control program (NACP) led by the National AIDS Control Organization (NACO) has kept IDUs at the forefront along with other key populations, in its efforts to prevent HIV. Given this, the objective of this study is to examine the association between IDUs’ degree of exposure to peer-led education sessions (under NACP) and their needle sharing practices in Haryana, India.

**Methods:**

The data for this study were drawn from a program monitoring system for the years 2009–2010 and 2010–2011. The relationship between IDUs’ background characteristics/injecting practices and degree of exposure to the program was assessed using chi-square and Student’s *t* tests. Generalized estimating equations (GEE) were used to examine changes in needle sharing practices over time by degree of exposure to peer-led education sessions. Further, the analysis was stratified by frequency of injecting drug use. All statistical analyses were conducted using STATA version 11.

**Results:**

The proportion of IDUs who shared needles substantially decreased from 2009 to 2011, particularly among those who attended three or more peer-led education sessions (49% vs 11%, *p* < 0.001) in a month. Further, subgroup analysis by frequency of injecting drugs demonstrates that this decline was significant among IDUs who injected frequently (adjusted odds ratio = 0.6, 95% confidence interval = 0.3–0.9, *p* = 0.043).

**Conclusion:**

The study results indicate that repeated peer-led outreach sessions are more effective than exposure to a single education session. Hence, HIV prevention programs must promote repeated peer contacts with IDUs every month (at least two meetings) in order to promote safe injecting practices and behavior change.

## Background

Injecting drug users (IDUs) are at high risk for blood-borne virus infections, including HIV, hepatitis B, and hepatitis C [1-3]. IDUs are also at risk of drug overdose and social instability [4] leading to higher morbidity and mortality in this group as compared to the general population [4]. In addition, high rates of anti-HCV and HBsAg coinfection have been recorded among HIV-infected IDUs [5]. For these reasons, the National AIDS Control Organization (NACO) in India has identified IDUs as a high-risk group (HRG) in need of HIV prevention interventions. In India, HIV prevalence is highest among IDUs at 7%, followed by men who have sex with men (4%), and female sex workers (3%) [6]. Recent estimates indicate that there are 177,000 IDUs in India [6]. While high HIV prevalence has been reported in the past among IDUs in the northeastern states of the country, similar results are now being reported from other states as well [7]. For example, in the state of Haryana, HIV prevalence among IDUs in 2011 was estimated to be 3%, ranging from 0% to 17% across districts, with two districts reporting rates of 10% or more (program data, Haryana 2011).

Among IDUs, one of the main routes of HIV transmission is through sharing of injecting equipment such as needles, syringes, and containers during drug use [8]. In 2006–2008, India reported the highest rates of needle-syringe sharing among IDUs in the Southeast Asian region [9]. In 2009, estimates of needle sharing among IDUs in India ranged from 62% in Chennai to 20% in the northeast [1,10,11]. In Haryana, a mapping exercise in 2003–2004 revealed that 48% of IDUs had shared needles [12]. Although needle sharing practices among IDUs have declined over the years due to NACO’s targeted intervention program, the practice of this risky injecting behavior continues to be high [11]. A variety of factors have led to the practice of needle sharing among IDUs in India, such as the fear of being harassed by the police and ‘anti-drug’ organizations for carrying sterile needles/syringes, the lack of sterile needles/syringes in drug dealers’ locales, limited access to pharmacy-sold needles/syringes, inadequate coverage of the needle and syringe program (NSP), non-availability of sterile needles/syringes in prisons, withdrawal symptoms that surpass health concerns, and poor mental health of IDUs [13].

In India, the HIV prevention response has been through NACO and the respective State AIDS Control Societies (SACS), under the Government of India’s National AIDS Control Program (NACP). The third phase of NACP has supported HIV prevention through peer-led outreach with a focus on key populations at high risk, including IDUs. Peer-led outreach has been defined as the facilitation of behavior change through the provision of information, training and/or support services to individuals by peers [14]. Peer-led outreach programs are based on the principle that peers can strongly influence an individual’s behavior [15,16] and that they share a level of trust and comfort with their peers that allow for more open discussions on sensitive topics [16-18]. Peers are familiar with the risks and concerns of the local population and can reach individuals who do not visit health care facilities [19]. Peer-led outreach programs are well-positioned to empower both the educator [20] and the target group by fostering a sense of solidarity and collective action [15,17,18,21,22].

While peer-led outreach has been adopted as a key strategy for behavior change among IDUs in Haryana, to our knowledge, no study to date has evaluated these efforts. Therefore, the objective of this study is to examine the association between IDUs’ degree of exposure to peer-led education sessions and their needle sharing practices in Haryana, India.

## Methods

### Study setting

In the state of Haryana, HIV prevention interventions for IDUs, including the Needle Syringes Program (NSP), were launched in three sites in 2008. Our analysis focuses on two of these sites: Amar Jyoti Foundation, Jind and Unnat Bharat Vikas, Panchkula (hereafter referred to as site 1 and site 2, respectively) and examines data covering a 2-year period (2009–2011). The third site was excluded from the analysis because of the lack of data on needle sharing practices. Of the two sites included in this study, one covers 300 IDUs while the other serves 512 IDUs. Both sites cover geographically clustered groups of IDUs living in rural and urban settings. In site 1, IDUs commonly inject a mixture of morphine/buperonorphine and Avil or a mixture of Phenergan and Avil, while in site 2, a mixture of Fortwin and Avil is typically injected. In both sites, IDUs commonly inject drugs in open, deserted areas like pits near railway tracks, slums behind factories, or public toilets. Injecting at chemist shops is less frequently reported.

### Peer-led outreach program

Peer educators provide IDUs a range of services through outreach in both sites. Peer educators are selected based on their leadership and communication skills; they are either chosen by fellow IDUs or volunteer their services. In addition to induction training, peer educators receive ongoing training to improve outreach activities.

Peer educators reach out to IDUs at ‘hot spots’ (common injecting areas), their residence, during group meetings at the program office or when IDUs visit drop-in centers. Each site has eight to ten peer educators, depending on the estimated size of the key population, and each peer educator is required to meet five to six IDUs a day during one-to-one or group education sessions. While one-to-one sessions generally last for 15–30 min, group sessions are longer (30–45 min), depending on the topics covered and the type of services provided during the session. Peer educators primarily counsel IDUs on behavior change including safe needle-syringe use and safe sex for the prevention of HIV and other sexually transmitted infections (STI). Services include the provision of disposable needles and syringes (sometimes on a daily basis), condom promotion and provision, STI/abscess management, oral substitution therapy and referral for detoxification, HIV testing, and anti-retroviral therapy for HIV-positive people.

In order to ensure quality services, peer educators are supervised by outreach workers, who visit them once a week in the field. Various indicators are used to evaluate the peer educators’ performance such as the number of meetings organized with IDUs, the number of IDUs contacted per week, and the services provided. Data on daily activities, including the number of beneficiaries met, the number of one-to-one and group sessions organized, the topics discussed and services provided, as well as behavioral information on IDUs such as needle sharing practices, are recorded in diaries and tracking sheets. These data are compiled by outreach workers on a weekly basis and by a counselor on a monthly basis and is shared with the SACS.

### Ethical considerations

To ensure the confidentiality of the respondents, data for this study did not include any personal identifiers. The Haryana State AIDS Control Society (HSACS), under the guidance of NACO, Department of AIDS Control, Government of India, provided general oversight and approval for the collection and analysis of routine programmatic data for examining the effect of the peer-led outreach program.

### Data

For this study, data on services provided to male IDUs and IDUs’ risky injecting practices during the years 2009–2010 and 2010–2011 were collected from peer educators’ diaries. In addition, sociodemographic data on IDUs were gathered from master registers, which were updated annually, as well as on an ongoing basis by program staff. Data from peer educators’ diaries, tracking sheets, outreach workers’ monthly registers, master registers, and outreach activity registers were cross-checked for accuracy. The first author also visited the program sites to validate the compiled data.

### Measures

The primary outcome measure considered in this paper was IDUs’ practice of needle sharing in the last 6 months (yes/no). Degree of exposure to peer-led sessions was the main independent variable. Based on the distribution of data and inputs from the program, we chose to categorize exposure as a dichotomous variable, with more than two meetings per month constituting ‘high exposure’ and two or fewer meetings constituting ‘low exposure.’ The background characteristics considered in this paper included age (measured as a continuous variable), education (had formal education, no formal education), occupation (laborer, regular employee, student, or unemployed), place of residence (rural, urban), and marital status (ever married, never married). Frequency of injecting drugs was measured as a continuous variable and was dichotomized as ‘low frequency’ (defined as fewer than two injections per day) and ‘high frequency’ (defined as two or more injections per day). The number of needle/syringes received per interaction with a peer educator was measured as a continuous variable and was log-transformed to normalize the distribution. Both sociodemographic characteristics and injecting practices were used as covariates in the multivariate analyses.

### Analysis

In order to examine the longitudinal effects of program exposure and needle sharing behavior, only IDUs who were followed up in the program between 2009 and 2011 were included in the analyses. We first assessed the relationship between IDUs’ sociodemographic characteristics, injecting practices, and degree of exposure to peer-led outreach sessions using chi-square and Student’s *t* tests, drawing on 2009–2010 data. Generalized estimating equations (GEE) were used to examine changes in needle sharing practices over time by degree of exposure to peer-led education sessions, adjusting for age, education, occupation, place of residence, marital status, program site, frequency of injecting drugs, and number of needles/syringes received per interaction with a peer educator. Further, the analysis was stratified by frequency of injecting drugs. All statistical analyses were conducted using STATA version 11.

## Results

Around one third of IDUs in site 1 were migrants from other states, of whom 19% were from Bihar, 17% were from Uttar Pradesh, 15% were from Punjab, and the rest were from other states, whereas the IDU population in site 2 mainly comprised the local population from the study state of Haryana. Among IDUs, 97% were from urban areas and 3% were from rural areas who visited urban areas to inject drugs (data not presented in tabular form). Of the 812 IDUs registered in the program, 102 were excluded from the analysis either due to loss to follow-up or because they had died during the study period.

Overall, the mean age of IDUs was 31 years; the majority had some formal education and were employed (Table 1). Fewer than half (44%) were ever married and three fourths resided in urban areas. No significant difference was observed in the degree of exposure to one-to-one peer education sessions in terms of age, marital status, education, and occupation. However, more rural IDUs than their urban counterparts had a high degree of exposure to peer-led education sessions (90% vs. 62%, *p* < 0.001); similarly, a larger proportion of those who were less frequent drug injectors as compared to those who injected drugs more frequently had high exposure to peer-led education sessions (80% vs. 55%, *p* < 0.001). The mean number of needles/syringes received per interaction with a peer educator was higher among those reporting less frequent interactions than those who had three or more interactions per month (mean 34 vs. 15).

**Table 1:** Background characteristics of IDUs by degree of exposure to one-to-one peer education sessions in Haryana, India

**Background characteristics**	**All participants (*n*** **= 710)**	**Low exposure**^**a **^**(*n*** **= 234)**	**High exposure**^**b **^**(*n*** **= 476)**	***p *** **value**
	***n *** **(%) or mean (SD)**	***n *** **(%) or mean (SD)**	***n *** **(%) or mean (SD)**	
Age (mean (SD))	31.0 (8.1)	31.3 (8.4)	30.9 (7.9)	0.416
Marital status				
Ever married	310 (43.7)	110 (35.5)	200 (64.5)	0.226
Never married	400 (56.3)	124 (31.0)	276 (69.0)	
Residence				
Urban	581 (81.8)	222 (38.2)	359 (61.8)	<0.001
Rural	129 (18.2)	12 (9.3)	117 (90.0)	
Education				
No formal education	140 (19.7)	42 (30.0)	98 (70.0)	0.392
Had formal education	570 (80.3)	192 (33.8)	378 (66.2)	
Occupation				
Laborer	316 (44.5)	98 (31.0)	218 (69.0)	0.156
Regular employee^c^	165 (23.2)	510 (30.3)	115 (69.7)	
Student	105 (14.8)	44 (41.9)	61 (58.1)	
Unemployed	124 (17.5)	42 (33.9)	82 (66.1)	
Program site				
Site 1	490 (69.0)	213 (43.5)	277 (56.5)	<0.001
Site 2	220 (31.0)	21 (9.6)	199 (90.0)	
Frequency of injecting drugs^d^				
Low	350 (49.3)	71 (20.3)	279 (79.7)	<0.001
High	360 (50.7)	163 (45.3)	197 (54.7)	
Number of needles/syringes received^e^ (mean (SD))	21.3 (17.4)	34.3 (20.1)	14.9 (11.5)	<0.001

Data represents IDU characteristics at entry into the intervention; *n* = 710. *p* value based on chi-square test of independence for categorical variables and Student’s *t* tests for continuous variables.

*SD* standard deviation.

^a^Attended two or fewer one-to-one peer education sessions a month.

^b^Attended more than two one-to-one peer education sessions a month.

^c^Self-employed/private/government employee.

^d^Low frequency, injected less than twice a day; high frequency, injected at least twice a day.

^e^Number of needles/syringes received per interaction with a peer educator.

As seen in Table 2, compared to 2009–2010, there was a considerable reduction in the proportion of IDUs reporting needle sharing practices in 2010–2011. Further, the decline in needle sharing practices over time was more among IDUs with high degree of exposure to peer-led education sessions as compared to IDUs with low degree of exposure (adjusted OR = 0.5, 95% CI 0.3–0.8). Additionally, the subgroup analysis by frequency of injecting practices reveals that this decline was statistically significant among IDUs who injected drugs frequently (adjusted OR = 0.6, 95% CI = 0.3 – 0.9, *p* < 0.043).

**Table 2:** Effect of degree of exposure to one-to-one peer education sessions on needle sharing practices among IDUs in Haryana, India

	**Low exposure**^**a**^		**High exposure**^**b**^		**Time×exposure to one-to-one peer education sessions**	***p *** **value**
	**2009–2010 (%)**	**2010–2011 (%)**	**2009–2010 (%)**	**2010–2011 (%)**	**Adjusted OR (95% CI)**	
All participants^c^ (shared needles (*n* = 710))	42.1	15.5	49.1	10.9	0.5 (0.3–0.8)	0.003
Subgroup analysis by frequency of injecting drugs						
Among low frequency IDUs^d,e^ (shared needles (*n* = 350))	42.3	8.1	50.7	8.2	0.7 (0.3–1.8)	0.492
Among high frequency IDUs^e,f^ (shared needles (*n* = 360))	42.8	19.9	45.4	13.9	0.6 (0.3–0.9)	0.043

*n* = 710; generalized estimating equation analysis.

*OR* odds ratio, *CI* confidence interval.

^a^Attended two or fewer one-to-one peer education sessions a month.

^b^Attended more than two one-to-one peer education sessions a month.

^c^Model adjusted for age, education, occupation, place of residence, marital status, program site, frequency of injecting drugs and number of needles/syringes received per interaction with a peer educator.

^d^Low frequency: injected less than twice a day.

^e^Model adjusted for age, education, occupation, place of residence, marital status, program site and number of needles/syringes received per interaction with a peer educator.

^f^High frequency: injected at least twice a day.

## Discussion

The results of this study indicate an association between IDUs’ degree of exposure to peer-led education sessions and safer injecting practices: the higher the degree of exposure to peer-led sessions, the lesser the likelihood of sharing of needles/syringes. This association is statistically significant, particularly among IDUs who inject drugs frequently. This finding provides evidence that high exposure to peer contact is effective in promoting safe injecting practices among IDUs in Haryana. This result is consistent with the literature, including a meta-analysis of data from India and other countries, that documents a positive association between exposure to HIV prevention interventions and safe injecting practices [11,22-26]. These results are also supported by an assessment of changes in IDUs’ HIV risk behaviors following scale-up of a targeted HIV prevention program in two northeastern states of India, where a strong association was observed between exposure to the intervention and a reduced likelihood of sharing needles [11].

The results of our study, combined with findings from other published research, make a strong case for the effectiveness of outreach interventions, including peer-led interventions, in facilitating positive changes in drug-related risk behaviors [11,16,18,22,23,25,27,28]. The study findings indicate a significant reduction in risky injecting practices, particularly among IDUs who inject frequently, and suggest that the program, through its microplanning approach, is reaching those most at risk for HIV.

Interestingly, we found that rural IDUs have greater exposure to peer education sessions, although IDUs in urban areas are typically more easily identified and contacted. However, this finding may reflect differences in the approaches used by peer educators in the two program sites or, alternatively, may indicate that IDUs in rural areas are more open to interacting with peer educators than others. Further, rural-based peer educators have fewer clients to visit as compared to their urban counterparts, allowing them more time for rapport building with their clients. Further research is needed to better understand this finding and whether additional strategies are needed to promote interaction between peer educators and IDUs in urban settings.

While this study provides important insights on the association between peer-led outreach efforts and changes in IDUs’ needle sharing practices, the findings should be interpreted in the light of certain limitations. First, we used data from program records maintained by peer educators, which may be subject to recording and reporting bias. Second, it is possible that IDUs who were more exposed to peer educator interactions were also more likely to provide socially desirable responses to questions on injecting risk practices. Third, our analysis is limited in geographical scope, and hence, our results cannot be generalized. Fourth, our analysis is limited to IDUs who were followed up by the program from 2009 to 2011; therefore, the effect of peer-led outreach on those lost to follow-up could not be captured. Fifth, the study does not have a control group; however, this study does not focus on whether peer-led outreach activity is effective but assesses the association between the degree of exposure to the program and the outcome. Finally, the introduction of other interventions in the study area, which also focused on people who inject drugs, may partially account for the observed reduction in risky injecting practices.

## Conclusion

Our analysis suggests that high exposure to peer-led outreach activities is an effective behavior change strategy for IDUs. It is crucial to reach IDUs who inject drugs frequently through targeted programs as they are more likely to share needles/syringes and thus increase their risk of HIV. In the study sites, project management through microplanning strategies, including identifying IDUs who are at high risk and least served, helped ensure repeated contact with IDUs. That said, it is critical that HIV prevention programs that monitor outcomes and impact as barriers to behavior change are dynamic. Moreover, it is unclear whether further declines in risk behaviors, such as needle sharing, may be achieved once peer-led outreach activities have saturated the population. Future studies should focus on identifying best practices as well as the differential impact of group versus individual outreach strategies, or a combination of both, among frequent injectors so that the strategy with the highest impact can be adopted in future programs targeting IDUs. Further, longitudinal studies of peer-led interventions, especially among new, emerging groups of IDUs, are needed to identify the dynamics of injecting practices and the effectiveness of peer-led prevention efforts.

## Competing interests

The authors declare that they have no competing interests.

## Authors’ contributions

BJ led the conceptualization and wrote the manuscript. SK assisted in the conceptualization and interpretation of study findings. SR and SS assisted with the conceptualization of analytic approach conducted in all analysis, interpretation of the results, and in the writing of the paper. VG and ND provided support and overall guidance. All authors read and approved the final submitted manuscript.
